# Performance appraisal of *Trichoderma viride* based novel tablet and powder formulations for management of *Fusarium* wilt disease in chickpea

**DOI:** 10.3389/fpls.2022.990392

**Published:** 2022-10-07

**Authors:** Prakash Chandra Pradhan, Arkadeb Mukhopadhyay, Randeep Kumar, Aditi Kundu, Neeraj Patanjali, Anirban Dutta, Deeba Kamil, Tusar Kanti Bag, Rashmi Aggarwal, Chellapilla Bharadwaj, P. K. Singh, Anupama Singh

**Affiliations:** ^1^ Division of Agricultural Chemicals, Indian Council of Agricultural Research (ICAR)-Indian Agricultural Research Institute, New Delhi, India; ^2^ Division of Plant Pathology, Indian Council of Agricultural Research (ICAR)-Indian Agricultural Research Institute, New Delhi, India; ^3^ Division of Genetics, (ICAR)-Indian Agricultural Research Institute, New Delhi, India

**Keywords:** *Trichoderma viride*, VOCs, tablet, seed treatment, *Fusarium* wilt, chickpea, IDM

## Abstract

In developing a *Trichoderma viride-*based biocontrol program for *Fusarium* wilt disease in chickpea, the choice of the quality formulation is imperative. In the present study, two types of formulations *i.e.* powder for seed treatment (TvP) and tablet for direct application (TvT), employing *T. viride* as the biocontrol agent, were evaluated for their ability to control chickpea wilt under field conditions at three dosages *i.e.* recommended (RD), double of recommended (DD) and half of recommended (1/2 RD). A screening study for the antagonistic fungi strains based on volatile and non-volatile bioassays revealed that *T. viride* ITCC 7764 has the most potential among the five strains tested (ITCC 6889, ITCC 7204, ITCC 7764, ITCC 7847, ITCC 8276), which was then used to develop the TvP and TvT formulations. Gas Chromatography-Mass Spectrometry (GC-MS) analysis of volatile organic compounds (VOCs) of *T. viride* strain confirmed the highest abundance of compositions comprising octan-3-one (13.92%), 3-octanol (10.57%), and 1-octen-3-ol (9.40%) in the most potential *T. viride* 7764. Further Physico-chemical characterization by standard Collaborative International Pesticides Analytical Council (CIPAC) methods revealed the optimized TvP formulation to be free flowing at pH 6.50, with a density of 0.732 g cm^-3^. The TvT formulation showed a pH value of 7.16 and density of 0.0017 g cm^-3^ for a complete disintegration time of 22.5 min. The biocontrol potential of TvP formulation was found to be superior to that of TvT formulation in terms of both seed germination and wilt incidence in chickpea under field conditions. However, both the developed formulations (TvP and TvT) expressed greater bioefficacy compared to the synthetic fungicide (Carbendazim 50% WP) and the conventional talc-based formulation. Further research should be carried out on the compatibility of the developed products with other agrochemicals of synthetic or natural origin to develop an integrated disease management (IDM) schedule in chickpea.

## Introduction

Chickpea (*Cicer arietinum* L.) is one of the most important legume crops (Sunkad et al., 2019) grown in the Mediterranean basin and worldwide. It is the third pulse crop in the world after dry bean (*Phaseolous vulgans* L.) and dry pea (*Pisum sativum* L.). In India, chickpea production occupies an area of 112 lakh hectares, resulting in approximately 116.20 lakh tonnes, with 1036 kg ha^-1^ in 2020-21. It covers approximately 38% of the area under pulse and contributes to around 50% of the total pulse production in India. Chickpea is an important source of protein for millions of people in developing countries. In addition to having high protein content (20-22%), chickpea is also rich in fiber, minerals (phosphorus, calcium, magnesium, iron, and zinc), and β-carotene (Yegrem, 2021).

Chickpea is exposed to several biotic and abiotic stresses among which pathogenic diseases are a major challenge. Chickpea is prone to attack by numerous pathogens, of which the most destructive is *Fusarium oxysporum* f. sp. *ciceris* causing wilt disease in chickpea crops (Golakiya et al., 2018). Wilt disease in chickpea caused by *F. oxysporum* f. sp. *ciceris* is one of the major causes of biotic stress to the crop. An average annual yield loss of 10-15% is reported due to this disease and under severe conditions, the damage may reach up to 100% (Navas-Cortes et al., 2000). Soil moisture stress and high-temperature conditions are conducive to soil-borne disease (Chand and Khirbat., 2009; Zaim et al., 2016; Alloosh et al., 2019). Carbendazim is the most widely used and recommended fungicide for the management of wilt disease in legumes (Srivastava et al., 2011). However, sole reliance on synthetic pesticides, due to injudicious and misuse is being discouraged globally, particularly in India, with more focus being given to greener options such as integrated disease management, and use of biopesticides, etc.

Biocontrol agent based products hold a major share of the biopesticides sector in agriculture. Biocontrol agents not only provide effective disease control but present safe and environmentally friendly options. The concept of biocontrol embodies the introduction of antagonists into cropping systems. A living multiplying biocontrol agent potentially provides continuous, non-chemical control of the pathogen. Moreover, chemical measures may establish an imbalance in the microbiological community *i.e.*, an unfavorable situation for the activity of beneficial organisms (Anusha et al., 2019). Therefore, direct application of antagonist would be a safer method for introducing microorganisms into the soil for biological control of soil-borne plant pathogens. It has been known for many years that biocontrol agents produce a wide range of antibiotic substances and that they parasitize other pathogenic fungi (Kumar et al., 2021). Among biocontrol agents, *T. viride* has been extensively reported as being effective at combating soil-borne diseases in field crops, including Fusarium wilt (Mei et al., 2019). A constraint in the large-scale adoption of bioagents despite their potential, is the lack of quality formulations with adequate cfu counts and viability. The most commonly reported and marketed products in this context are wettable powder formulations that employ talc as a carrier (Ramanujam et al., 2010). In India, wettable powder of *T. viride* (1% WP) is recommended for the management of chickpea wilt (CIBRC, 2022). Poor load of cfu in stored WP formulations coupled with the poor coating efficacy of dry powders and dust hazards necessitate precision formulation approaches for biocontrol products. Furthermore, WP or dust powder for seed treatments employs a carrier only. Since the performance of *Trichoderma* is positively related to moisture availability in the zone of its application, the addition of adjuvants like carbon sources and moisture enriching polymers can provide additional benefits for biocontrol (John et al., 2011). As per the Central Insecticides Board and Registration Committee (CIBRC, India), an authentic biocontrol formulation of *Trichoderma* spp. should have 2×10^6^ cfu g^-1^ of formulation at the time of its application in the field (CIBRC, 2011).

The present work reports on the development, characterization, and performance assessment of two innovative formulations of *T. viride*, namely, tablets (TvT) and dustable powder for seed coating (TvP), employing biopolymer, clay, neem leaf powder as an environmentally benign alternative to conventionally used synthetic fungicides to manage *Fusarium* wilt in chickpea. Excipients have previously been used to provide a moist environment in the formulation’s application zone. With this in mind, the objectives of the present study were (1) to develop and characterize a novel tablet formulation of *T. viride*; (2) to evaluate the potential of the developed tablets against chickpea wilt under *in vivo* conditions; and (3) to ascertain the dose-response and relative performance of the optimized tablet formulation with a powder formulation under field conditions to provide an alternative to the conventional talc-based WP formulation and synthetic fungicide, Carbendazim 50% WP.

## Materials and methods

### Culture medium and reagents

Dehydrated potato dextrose agar medium (PDA) and potato dextrose broth (PDB) were procured from HiMedia^®^ laboratories (Mumbai, India) and used as culture media for the pathogen, *F. oxysporum* f. sp. *ciceris* and the biocontrol agent *T. viride*, obtained from Indian Type Culture Collection (ITCC), ICAR-Indian Agricultural Research Institute, New Delhi, India. Neem leaves were collected from trees on the institute premises, dried, and powdered to around 100 -240 mesh size (62.5 -150µ) before use. Biopolymer (pH 6.0-6.5; water absorption capacity 80 g g^-1^ was purchased from the local market and used. Aluminosilicate clay mineral (80-90% silica, 2-3% alumina) was obtained from Casa De Amor, Madhya Pradesh, India. Bentonite clay (Al_2_O_3_. 4 SiO_2_. H_2_O; pH of 2% suspension in water: 9.00-10.50) was obtained from Hi-Media Laboratories Pvt. Limited, Mumbai, India. The xerogel used was sugarcane bagasse-based biopolymeric grafted and crosslinked polyacrylate hydrogel composite prepared in our laboratory (Particle size: 120 -200 mesh size (75 -125µ), WAC 600 g g^-1^). Carboxymethyl cellulose sodium salt (CMC) (LR grade; viscosity: 1100-1900 cps as per label claim) was purchased from Merck^®^ Life Science Pvt. Ltd., Mumbai, India.

### Pathogen and antagonistic fungi

Pure culture of *F. oxysporum* f. sp. *ciceris* in slants was obtained from the Indian Type Culture Collection (ITCC), Division of Plant Pathology, ICAR-Indian Agricultural Research Institute, New Delhi, India. Pure cultures of five *T. viride* strains (ITCC 6889, ITCC 7204, ITCC 7764, ITCC 7847, ITCC 8276) were also obtained from ITCC. The pathogenic and biocontrol fungi were sub-cultured for one week and four days in Petri plates using potato dextrose agar (PDA) and mass produced on sterilized sorghum (*Sorghum bicolor*) grains (Khan et al., 2001).

For mass culturing, sorghum grains were soaked in distilled water for 12h, strained, and filled into various conical flasks (500 mL). The flasks containing sorghum grains were autoclaved for two subsequent days at 1.1 kg cm^-2^ for 30 min and inoculated with a 7-day-old culture of *T. viride* ITCC 7764. The flasks were incubated at 25 ± 1 °C for 15 days. Well-colonized sorghum grains were then taken out from the flask, dried at room temperature, and finally made into a fine powder.

### *In vitro* evaluation of *T. viride* strains against *F. oxysporum*

*T. viride* strains (ITCC 6889, ITCC 7204, ITCC 7764, ITCC 7847, and ITCC 8276) were tested *in vitro* for antagonistic activity against *F. oxysporum*. To select the most potential biocontrol *T. viride* strain, two-way assays namely, volatile and non-volatile *in-vitro* antifungal methods were performed.

### Effect of volatiles of *T. viride* on growth of *F. oxysporum*

The test was carried out using the inverted plate technique as described by Sheoran et al. (2015). Briefly, the upper lid of the PDA plate was inoculated with the pathogen and the lower lid with a strain of *T. viride* separately under aseptic conditions. The two lids were taped together to facilitate the exposure of the pathogen to volatile organic compounds released by the biocontrol fungi, then incubated at 27 ± 1°C for seven days. The experiment was conducted in triplicate and negative control was maintained with *F. oxysporum*. Relative radial growth and inhibition (%) were computed with reference to control, according to the equation suggested by Tapwal et al. (2011):


N=((A-B)/A)*100

where N is growth inhibition percentage, A is the average diameter of the control colony and B is the average diameter of the treatment colony

### Effect of non-volatile components of *T. viride* on growth of *F. oxysporum*

The test was carried out using the method reported by Choi and Ahsan (2022). Briefly, the *T. viride* cultures were inoculated in conical flasks (250 mL) containing sterile potato dextrose broth (PDB) and incubated at 27 ± 1°C for two weeks. The culture was filtered through a micropore filter (0.22 µ) and filtrates were collected in a sterile flask. The culture filtrate was added to the molten PDA medium to obtain a final concentration of 10% (v v^-1^). Upon its solidification, a 5 mm disc of the pathogen was inoculated. Negative control plates were also maintained. The radial growth (mm) of colonies was recorded and inhibition (%) was calculated relative to control.

### GC-MS analysis of volatile organic components

GC-MS analysis was carried out to characterize VOCs from potential *T. viride* strains using a 5590C Gas Chromatograph, equipped with a Mass Spectrometer (Agilent Technologies^®^, USA). Volatiles were separated through an HP-5MS capillary column (30 m × 0.25 µm; 0.25 µm). The VOCs were allowed to adsorb on Tenax TA polymer (80-100 mesh) for 8h and then extracted in GC-MS grade hexane (Kumar et al., 2021). Samples were injected (1µL, each) through an autoinjector following the split mode, 1:10. Helium (>99.9% purity) was used as carrier gas with a flow rate of 0.75 mL min^-1^ and pressure of 15 psi. Oven temperature ramping started at 40°C and raised at the rate of 3°C min^-1^ to reach 130°C and held for 2 min. Again, the temperature was raised at the rate of 5°C min^-1^ to reach 200°C and held for 2 min. Finally, the oven temperature was increased at the rate of 10°C min^-1^ to reach 300°C. The total run time was 58 min. Besides, other mass acquisition parameters were tuned before analysis and fixed with the ion source temperature of 200°C, transfer line temperature of 200°C, solvent delay of 3 min, and E.M voltage 1420 V. The scanning rate per second was fixed with the mass range m/z 50-550 amu. Identification of volatile compounds was carried out based on their retention indices (RI) and calculated using the homologous series of *n*-alkanes (C_9_-C_24_) before being matched with the library database of the NIST (National Institute of Standards and Technology) Version 3.02 (Keerthiraj et al., 2021).

### Compatibility evaluation of neem leaf powder with *T. viride* and *F. oxysporum*

The random choice of formulation adjuvants may result in poor efficacy. For the selection of the neem leaf powder as filler material for the development of the tablet and powder formulations of *T.viride*, it is imperative to generate information on its compatibility with the biocontrol agent as neem leaf powder is known to be rich in various secondary metabolites having pesticidal properties (Patil et al., 2018). Therefore, *in vitro* studies were performed to assess the compatibility of neem leaf powder with the biocontrol agent and the biocontrol potential singly and in combination with the biocontrol agent. The PDA culture media was prepared as described by Bunbury-Blanchette and Walker (2019).

### Test concentrations of neem leaf powder

The compatibility of *T. viride* was tested with neem leaf powder under laboratory conditions at test concentrations of 800µg/mL, 400 µg/mL, and 200 µg/mL. Test concentrations were prepared using acetone as solvent. At these concentrations of neem leaf powder, TvT or TvP formulations at double the recommended dose, the recommended dose, and half of the recommended dose were applied for *in vivo* experiments.

### Compatibility evaluation of neem leaf powder with *T. viride* and *F. oxysporum*

A definite volume of neem leaf powder in acetone was thoroughly dispersed into a sterilized PDA medium to furnish different test concentrations of 800 -200 µg/mL. Acetone (1 mL) solution was taken as control. Media containing neem leaf powder (20 mL) was poured into each Petri dish. The culture (5 mm diameter disc) of both the fungi was placed separately in the center of each dish. Three replicates were maintained for each treatment along with the negative control and the experiment was repeated twice. Petri dishes were incubated in a BOD incubator at 28 ± 1°C. Data were recorded after 3 days and 7 days in the case of *T. viride* and *F. oxysporum*, respectively.

### Preparation of biocontrol formulations

The best performing *T. viride* strain (Tv, ITCC 7764) was used as an active ingredient (*a.i.* Biomass) to develop two novel biocontrol formulations, the tablet for direct application (TvT) and powder for seed treatment (TvP). To optimize the composition for TvT, a standardization study was conducted at different concentrations of ingredients. The tablet formulation for direct application (TvT) was developed by using *T. viride* spores as a biocontrol agent, carboxy methyl cellulose (CMC), neem leaf powder as fillers, xerogel, and bentonite as carriers. The tablets were prepared by hand operated tablet making machine at room temperature (26-28 °C). The present study used an optimized composition of powder formulation named TvP2, which was previously developed for seed treatment by our laboratory for the comparative evaluation of the bioefficacy of the tablet (TvT) formulation in managing chickpea wilt disease under field conditions. The excipients used for the development of the TvP formulation included a pre-standardized ratio of biopolymer, neem leaf powder, xerogel, and a porous clay mineral carrier other than talc. The general procedure involved a pre-standardized sequence of mixing the fungal biomass with the carrier and the excipients under ambient conditions. The developed compositions were stored in air-tight glass bottles at 25°C. The composition variations of the prepared formulations are depicted in **Table 1**. The optimized TvP formulation has shown potential bioefficacy against *F. oxysporum* f. sp. *ciceris* under *in vivo* conditions, as reported elsewhere. Briefly, the application of TvP formulation at recommended doses resulted in 3.33% wilting in chickpea under *in vivo* conditions. However, the bioefficacy of the TvP formulation under field conditions has yet not been evaluated. Following on from this previous research, the present study undertook a comparative evaluation of the TvT and TvP formulations (previously developed in our laboratory) under field conditions.

**Table 1:** Compositions of developed TvT and TvP formulations.

Code	Bentonite (% w w^-1^)	CMC (% w w^-1^)	Neem leaf powder (% w w^-1^)	Optimized xerogel (% w w^-1^)
TvT1	80.94-81.99	2.5-3.5	9.8-10.3	4.7-5.5
TvT2	83.98-84.99	2.5-3.5	9.8-10.3	2.0-2.4
TvT3	78.99-79.99	2.5-3.5	9.8-10.3	6.9-7.3
TvT4	86.50-86.99	2.5-3.5	4.8-5.2	4.8-5.2
TvT5	75.99-76.99	2.5-3.5	14.5-15.2	4.8-5.2
TvT6	83.00-83.49	1.3-1.7	9.8-10.3	4.7-5.5
TvT7	80.50-80.99	1.8-2.1	9.8-10.3	4.7-5.5
TvT8	71.50-71.99	12.5-13.5	9.8-10.3	4.7-5.5
TvT9	86.70-86.99	0.90-1.20	7.0-7.3	4.8-5.2
	**Neem leaf powder** **(% w w^-1^)**	**Biopolymer** **(% w w^-1^)**	**Porous clay mineral** **(% w w^-1^)**	**Optimized xerogel** **(% w w^-1^)**
TvP2	28.38-29.03	20.97-21.62	20.97-21.62	28.38-29.03

### Physico-chemical characterization of optimized TvP and TvT formulations

The pH, flowability, density and sieve analysis of the optimized TvP formulation (TvP2) were carried out by following MT 75, MT 44, MT 3.2.1, and MT 59.1 of the standard CIPAC guidelines. We undertook a standardization study of TvT composition at different levels of ingredients as a function of physicochemical characteristics, the procedure for which is described below.

### pH

For TvT, the pH of each composition was measured by using the CIPAC method MT 75. Briefly, around 1 g of sample tablet was put into a 250 mL glass beaker and completely dissolved in 100 mL of water. After the complete disintegration of the tablets, the beaker was stirred for 1 min and the pH of the supernatant was recorded.

### Visible extraneous matter

The tablets were immediately weighed and then kept undisturbed for 1h on Petri plates. After one hour, the tablets were whisked thoroughly and then the extraneous formulation residue left on the plate was weighed and recorded as grams per tablet.

### Moisture content

The moisture content of the developed tablets was measured gravimetrically (Sun et al., 2020). The pre-weighed (W1) tablets were placed in an oven at 60 °C and weighed periodically until they attained a constant weight (W2). The percent moisture content of the TvT compositions was calculated using the following formula:


%Moisture content=(W1−W2/W1)*100

### Disintegration time

The time for complete disintegration of the tablets was determined by the CIPAC method MT 197. Briefly, one tablet was added to standard hard water D (1800 mL) and gently stirred until it completely disintegrated. The suspension was passed through a 2000 µ sieve. The time taken for complete disintegration using standard procedure was recorded.

### Tablet integrity

Tablet integrity was determined visually by observing whether any tablet of the table pack prepared in each batch was broken or not.

### Density

First, the volume of the tablet was determined using the vernier caliper scale, followed by density calculation as per the standard formula, d=m/v (Eiliazadeh et al., 2003).

### *In-vivo* assessment of optimized TvT formulation against wilt disease in chickpea

A pot experiment was conducted in the net house of the Division of Plant Pathology, ICAR-IARI, New Delhi, India. The soil of the sick plot (*Fusarium oxysporum* f. sp. *ciceris* infested) of the institute farm was used (pH-7.8, EC- 5.6 dS m^-1^, organic matter 0.4%) to fill the pot (18 cm×15 cm) dimension. In total, 2kg of soil was used to fill each pot. The treatment details for the pot experiment are depicted in **Table 2**. Briefly, six treatments with three replications per treatment were employed for the experiment. A total of 10 seeds per pot (for each replication) were sown to record different observations. The parameters recorded to assess the performance of formulation were the wilting incidence and seed germination. The percentage of germination of seeds without infection was 94% as received as the foundation seeds from the Division of Genetics, ICAR-IARI, New Delhi, India. In pot experiments, the seed emergence was recorded 21 days after sowing (DAS). Observations on the number of plants wilted in each pot were recorded at 30, 45, and 60 DAS. The plants that showed dropping petioles, rachis, and leaflets without any external rotting in the roots, but dark brown discoloration of the internal xylem were considered wilted (Nene et al., 2012).

**Table 2:** Treatment details from bio-efficacy studies of developed formulations under pot and field experiments.

S. No.	Treatments*	Treatment details
1	T1	Absolute control
2	T2	Carbendazim 50% WP
3	T3	Talc formulation
4	T4	Test formulation (TvT) at RD
5	T5	Test formulation (TvT) at DD
6	T6	Test formulation (TvT) at HD
7	T7	Test formulation (TvP) at RD
8	T8	Test formulation (TvP) at DD
9	T9	Test formulation (TvP) at HD

*[Treatments for pot experiment: T1-T6; Treatments for field experiment: T1-T9].

(RD, Recommended dose; DD, Double dose; HD, Half dose; TvT, Tablet for direct application; TvP, Powder for seed treatment).

The causal agent of wilt incidence was confirmed after re-isolation of the pathogen from the infected root and stems of chickpea plants. The wilt incidence (%) was calculated based on the initial plant count and the total number of wilted plants in each pot. The disease was monitored for 6-8 weeks and assayed as the total percentage of plants showing any wilt symptoms due to the pathogen (yellowing and dropping of leaves, vascular discoloration, and wilting). The stem sections of wilted plants were surface disinfested in 0.5% sodium hypochlorite and plated on a Pentachloronitrobenzene (PCNB) medium to confirm the presence of the wilt pathogen. The stem sections of asymptomatic plants were also plated at the conclusion of the experiment to evaluate potential pathogen infection.

### Comparative bio-efficacy assessment of TvT and TvP against wilt disease under field condition

The two bioformulations (TvT and TvP) were assessed for their performance in affecting wilting incidence, inhibition of seed germination, and mortality of chickpea seedlings caused by the pathogen under *Fusarium* wilt sick field conditions. The field experiment was conducted at the institute farm of ICAR-IARI, New Delhi, India, during the rabi season (2021-2022). The natural abundance of Fusarium in the sick field was 2× 10^7^ cfu g^-1^ of soil at the time of sowing. A randomized block design with nine treatments along with three replications per treatment was applied. Moderately susceptible chickpea variety, Pusa-372 was taken for the experiment. The treatment details for the field experiment were used to check the comparative bio-efficacy of the developed TvT and TvP formulations against *Fusarium* wilt in chickpea have been depicted in **Table 2**. The crop was sown with 30×10 cm spacing having a gross plot size of 9.0×9.0 m and a net plot size of 2.0×0.6 m. The seed rate used was 60 kg ha^-1^. Seeds were sown in microplots in rows (20 seeds per row, 2 rows per microplot), *i.e.* a total of forty seeds were sown in each microplot of each treatment. The pathogen population in the sick field was determined at the pre-sowing stage. Seed germination (%) was recorded two weeks after sowing and computed based on the number of germinated seeds (emerged seedlings) out of the total number of seeds sown in a microplot (40 seeds).

The seed emergence was recorded 21 days after sowing (DAS). Observations on the number of plants that wilted in each microplot were recorded at 30, 45, and 60 DAS. The wilting incidence (%) was calculated based on the initial plant count and the total number of wilted plants in each microplot. At maturity, seed weight and grain yield were recorded for each treatment.

### Statistical analysis

The *in vitro* bioefficacy of the selected *T. viride* strains using volatile and non-volatile methods along with the efficacy of the neem leaf powder singly and in combination with the antagonistic fungi at three different concentrations against *F. oxysporum* were analyzed by comparing mean differences using a completely randomized design by Duncan Multiple Range test at p< 0.05. Different test parameters such as germination (%), relative disease incidence (%), and yield parameters such as percent yield, shoot length, root length, and shoot dry matter in test treatments were analyzed by randomized complete block design using a one-way analysis of variance (ANOVA) and grouped by Duncan Multiple Range test at p< 0.05 using PROC GLM procedure of SAS 9.3 (SAS Institute, Cary, North Carolina, USA).

## Results

### *In-vitro* evaluation of *Trichoderma viride* strains against *Fusarium* wilt pathogen

**Figure 1:**
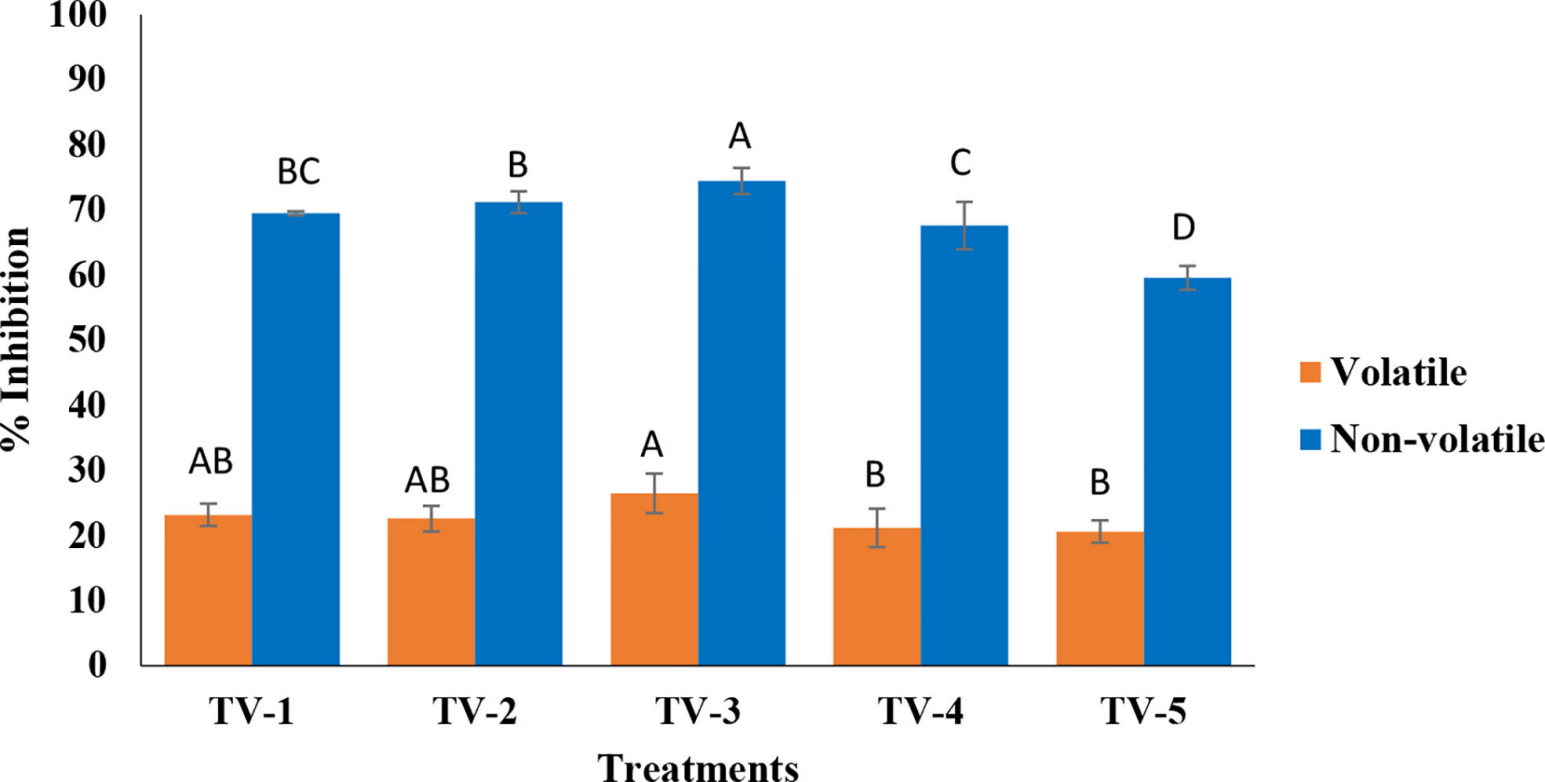
Assessment of secondary metabolites of *T. viride* against pathogenic wilt causing fungus, *F. oxysporum* f. sp. *ciceris* by volatile and nonvolatile methods. (Tv1- ITCC 6889, Tv2- ITCC 7204, Tv3- ITCC 7764, Tv4- ITCC 7847, Tv5- ITCC 8276, Control-without *Trichoderma* strain). (For each method, treatment bars having at least one letter common are not statistically significant using Duncan’s Multiple Range Test at p<0.05, n=3, error bars represent standard deviations).

Based on the *in-vitro* bio-efficacy assessment of five strains of *T. viride* strains against *F. oxysporum* f. sp. *ciceris* suggested the suitability of *Trichoderma* (ITCC 7764) as a potential biocontrol agent to manage wilting in chickpea (**Figures 1**, **S1**, and **S2**). In the volatile assay, the volatile components produced by all the strains significantly inhibited the pathogen but did so relatively less than the non-volatile method. Pathogen growth inhibition ranged between 21.15-26.45% with the volatiles, out of which TV-3 (26.45%) recorded the maximum inhibition followed by TV-1 (23.15%), TV-5 (22.58%), TV-2 (22.56%) and TV-4 (21.15%). Furthermore, all the strains of *Trichoderma* were assessed to inhibit the radial growth of the *F. oxysporum*, attributed to the effect of non-volatile organic compounds. Strains TV-3 (74.45%) and TV-2 (71.2%) recorded maximum inhibition of mycelial growth, significant potential over the other strains. Strains TV-1 (69.5%), TV-4 (67.58%), and TV-5 (59.54%) performed moderately to control the growth of the pathogen.

### Characterization of VOCs

Volatile metabolites of the potential *T. viride* strains were characterized using GC-MS, which displayed identification of a total of twenty-seven VOCs, listed as per their elution from the HP-5MS column (**Table 3**). Total Ion Chromatogram (TIC) of *T. viride* 7764 showed the maximum number of peaks corresponding to nineteen components. Among the identified VOCs of *T. viride* 7764, octan-3-one (13.92%), 3-octanol (10.57%) and 1-octen-3-ol (9.40%) were most abundant. Other major components were 6-methyl-bicyclo-octan-7-ol (5.34%), 9-octadecenoic acid-methyl ester (4.98%), hexadecanoic acid-methyl ester (4.17%), methyl-6-arachidonate (3.46%), 2-Mthyl-butanol (1.79%), 2-ethyl-hexanal (1.72%), and heptadecanoic acid-methyl ester (1.40%). Besides, 2-pentyl-furan (0.91%), 2-nonanone (0.83%), phenylethyl alcohol (0.72%), 3-methyl-butanal (0.64%), 2,4-Decadienal (0.58%), 2-undecanone (0.39%), 3-hexene-1-ol (0.33%), 2-heptyl-furan (0.26%), and octacosanol (0.18%) were identified as minor components of *T. viride* 7764.

**Table 3:** Chemical composition of the volatile organic compounds of potential *trichoderma viride* strains as analysed in gc-ms.

S No.	^a^Compound	^b^RI^exp^	^c^Area %					^d^Detection
			*T. viride* 6889	*T. viride* 7204	*T. viride* 7764	*T. viride* 7847	*T. viride* 8276	
1	2-Methylpropanal	554	0.37 ± 0.12	0.19 ± 0.00	–	0.24 ± 0.10	–	NIST, RI
2	3-Methyl-butanal	648	1.09 ± 0.39	–	0.64 ± 0.15	–	–	NIST, RI
3	2-Pentanone	682	–	–	–	0.73 ± 0.12	0.14 ± 0.00	NIST, RI
4	2-Mthyl-butanol	737	1.60 ± 0.21	0.36 ± 0.12	1.79 ± 0.28	–	–	NIST, RI
5	3-Hexene-1-ol	855	tr	1.02 ± 0.00	0.33 ± 0.05	2.25 ± 0.31	7.29 ± 1.14	NIST, RI
6	2-Heptanone	891	–	0.46 ± 0.09	tr	1.78 ± 0.42	–	NIST, RI
7	2-Ethyl-hexanal	953	2.06 ± 0.53	1.11 ± 0.20	1.72 ± 0.37	2.29 ± 0.36	6.64 ± 0.88	NIST, RI
8	1-Octen-3-ol	984	3.35 ± 0.19	7.42 ± 1.41	9.40 ± 1.71	–	0.98 ± 0.10	NIST, RI
9	Octan-3-one	986	5.27 ± 0.67	6.79 ± 0.78	13.92 ± 2.65	tr	tr	NIST, RI
10	2-Pentyl-furan	990	–	tr	0.91 ± 0.12	–	–	NIST, RI
11	3-Octanol	997	0.74 ± 0.12	11.06 ± 2.26	10.57 ± 1.33	0.63 ± 0.12	0.15 ± 0.00	NIST, RI
12	2-Nonanone	1094	–	–	0.83 ± 0.15	–	–	NIST, RI
13	Phenylethyl alcohol	1120	–	0.23 ± 0.00	0.72 ± 0.00	1.54 ± 0.29	0.27 ± 0.12	NIST, RI
14	2-Methyl-1-undecene	1182	1.02 ± 0.16	tr	–	–	tr	NIST, RI
15	2-Heptyl-furan	1202	0.12 ± 0.00	–	0.26 ± 0.10	–	–	NIST, RI
16	2-Undecanone	1289	–	3.47 ± 0.98	0.39 ± 0.00	0.63 ± 0.12	–	NIST, RI
17	2,4-Decadienal	1318	tr	0.60 ± 0.12	0.58 ± 0.17	2.92 ± 0.11	4.82 ± 0.55	NIST, RI
18	Dodecane	1461	5.32 ± 0.92	–	–	9.38 ± 2.17	17.44 ± 3.48	NIST, RI
19	2,4-Dodecadienal	1515	–	0.41 ± 0.12	tr	1.18 ± 0.21	0.54 ± 0.19	NIST, RI
20	1-Hexadecene	1598	–	0.38 ± 0.00	tr	–	0.12 ± 0.00	NIST, RI
21	6-Methyl-bicyclo-octan-7-ol	1720	tr	1.27 ± 0.34	5.34 ± 0.96	–	–	NIST, RI
22	Hexadecane		–	–	tr	3.22 ± 0.15	1.46 ± 0.58	NIST, RI
23	Hexadecanoic acid-methyl ester	1925	1.18 ± 0.00	3.92 ± 0.96	4.17 ± 0.82	–	0.93 ± 0.25	NIST, RI
24	Heptadecanoic acid-methyl ester	2030	0.24 ± .12	2.58 ± 0.14	1.40 ± 0.23	1.51 ± 0.12	1.24 ± 0.69	NIST, RI
25	9-Octadecenoic acid-methyl ester	2105	–	1.34 ± 0.13	4.98 ± 0.17	–	–	NIST, RI
26	Methyl-6- arachidonate	2208	–	1.00 ± 0.19	3.46 ± 0.45	–	–	NIST, RI
27	Octacosanol	3110	0.67 ± 0.00	–	0.18 ± 0.00	tr	0.35 ± 0.00	NIST, RI

^a.^Compounds are listed in order of their elution from an HP-5MS column.

^b.^Retention index (RI^exp^) on HP-5MS column, experimentally determined using homologous series of C_8_-C_30_ alkanes.

^c.^Relative area % values are expressed as means ± SD.

^d.^Detection: RI, NIST (matching with the mass library), and mass fragments.

* tr-trace<0.1%.

*T. viride* 7204 produces a high amount of 3-octanol (11.06%), 1-Octen-3-ol (7.42%), and octan-3-one (6.79%). Other prominent components were identified as hexadecanoic acid-methyl ester (3.92%), 2-undecanone (3.47%), heptadecanoic acid-methyl ester (2.58%), 9-octadecenoic acid-methyl ester (1.34%), 6-methyl-bicyclo-octan-7-ol (1.27%), 2-ethyl-hexanal (1.11%), 3-hexene-1-ol (1.02%), and methyl-6-arachidonate (1.00%). Similarly, the GC-MS chromatogram of volatiles of *T. viride* 6889 exhibited a relatively smaller number of components than the previous two strains. Structural long chain hydrocarbon dodecane (5.32%) was the major compound followed by octan-3-one (5. 27%). A similar observation of the high content of dodecane was estimated in *T. viride* 7847 (9.38%) and *T. viride* 8276 (17.44%). Except dodecane, other major volatile components of *T. viride* 7847 were hexadecane (3.22%), 2,4-decadienal (2.92%), 2-ethyl-hexanal (2.29%), 3-hexene-1-ol (2.25%). Likewise, 3-hexene-1-ol (7.29%), 2-ethyl-hexanal (6.64%), 2,4-decadienal (4.82%), hexadecane (1.46%), and heptadecanoic acid-methyl ester (1.24%) were identified in *T. viride* 8276.

### Compatibility evaluation of neem leaf powder against *T. viride*

The *in-vitro* efficacy of neem leaf powder was assessed against *T. viride* using the poisoned food technique. The result suggested that there was no inhibition of the biocontrol agent upon the addition of neem leaf powder in the formulation at all three test dosage levels. This result confirmed that neem leaf powder could be used as an adjuvant to *T. viride*- based formulation.

### Compatibility evaluation of neem leaf powder against *F. oxysporum*

The neem leaf powder was further assessed to diagnose the effect on the target pathogen and revealed significant inhibition of the test fungus, *F. oxysporum i.e.* 18.6%, 12.8%, and 5.8% at double, recommended, and half doses, respectively. The result signifies that the neem leaf powder can be utilized in the formulation to enhance the synergistic effect against the target pathogen (**Table 4**).

**Table 4:** Effect of Neem Leaf powder and *Trichoderma viride* + Neem Leaf Powder on *Fusarium oxysporum* f. sp. *ciceris* (*in vitro*).

Dose	% Inhibition*	
	Neem leaf powder	*Trichoderma viride* + neem leaf powder
Double dose (800 ppm)	18.6 ± 2.9^A^	50.6 ± 3.4 ^A^
Recommended dose (400 ppm)	12.8 ± 1.2 ^B^	34.9 ± 4.5 ^B^
Half dose (200 ppm)	5.8 ± 1.3 ^C^	16.5 ± 1.1 ^C^
CV (%)	13.97	2.94

*Means with at least one letter common are not statistically significant using DUNCAN’s Multiple Range Test at p<0.05, n=3.

### Effect of *T. viride* and neem leaf powder on *F. oxysporum*

To assess the synergistic effect of neem leaf powder and *T. viride*, both the components were assessed in dual culture technique against the target pathogen. The result suggested that there was direct synergism in the combined application of neem leaf powder and *T. viride*. Upon application of respective concentrations at three test dosage levels (double, recommended, and half dose), 50.6%, 34.9%, and 16.5% inhibition of the test fungus were observed, respectively (**Table 4**).

### Physico-chemical properties of optimized TvP and developed TvT formulations

The preparation of optimized TvP and developed TvT formulations are outlined in **Table 1**. The different physicochemical properties of the developed optimized TvP formulation (TvP2) and developed TvT formulations used in this study are presented in **Table 5**. Briefly, the optimized TvP2 formulation was found to be free flowing, with a pH of 6.50 and a density of 0.732 g cm^-3^.

**Table 5:** Effect of formulation compositions on the physico-chemical characteristics of developed TvT and TvP formulations.

TvT formulations	Parameters													
	pH	Visible extraneous matter(g tablet^-1^)		Moisture content(%)		Disintegration time(min)				Tablet integrity			Density (g cm^-3^)	
**TvT1**	7.16	0.0046		7.55		22.50				No broken tablets			0.0017	
**TvT2**	7.33	0.0044		10.07		24.00				No broken tablets			0.0016	
**TvT3**	7.24	0.0082		9.77		26.50				No broken tablets			0.0017	
**TvT4**	7.34	0.0056		9.97		23.50				No broken tablets			0.0017	
**TvT5**	7.45	0.0062		9.31		25.00				No broken tablets			0.0016	
**TvT6**	7.32	0.0083		9.84		26.50				No broken tablets			0.0017	
**TvT7**	7.41	0.0055		9.76		23.50				No broken tablets			0.0016	
**TvT8**	7.21	0.0043		9.48		26.00				No broken tablets			0.0015	
**TvT9**	7.48	0.0070		9.88		24.50				No broken tablets			0.0018	
**TvP formulation**	**Parameters**													
	**pH**	**Flowability**	**Density** **(g cm^-3^)**		**% Retention of powder (mesh size wise)**									
					**BSS 5**		**BSS16**		**BSS** **30**	**BSS60**	**BSS 100**	**BSS 120**	**BSS 240**	**BSS 325**
**TvP2**	6.50	Free-flowing	0.732		0			1.1	4.4	14.6	1.9	3.6	41.9	0.2

The physicochemical characteristics of the prepared TvT formulations for optimization of the most suitable composition revealed variations depending on the formulation compositions. The pH is considered one of the most important parameters of any bioformulation. The recorded pH of the prepared tablets was found to be slightly alkaline having a nominal value from 7.16 to 7.48. The positive effect of the amount of bentonite clay on pH increment was recorded. The composition TvT9 with the maximum amount of bentonite clay (86.70-86.99%, w w^-1^) revealed the highest pH value of 7.48. The amount of visible extraneous materials in the prepared tablets was in the range of 0.0043-0.0083 g per tablet. The percent moisture content of the developed tablets was found to be in the range of 7.55% (TvT1) to 10.07% (TvT2). No specific pattern was observed in the changing of the moisture content of the prepared tablets due to variation in compositions. The complete disintegration time is an important factor for the efficacy of the tablets intended for direct application. The longer time of disintegration may affect the desired bio-efficacy of the developed product. In our case, the prepared tablets showed 22.5 min to 26.5 min time for complete degradation of a single tablet. The lowest disintegration time (22.5 min) was recorded in the TvT1 formulation. All the prepared tablets irrespective of their composition were found to be non-breakable. The density of the tablets varied from 0.0015 g cm^-3^ (TvT8) to 0.0018 g cm^-3^ (TvT9). There was not much variation in the density of the developed tablets. The variations in xerogel, neem leaf powder, and CMC content in the composition did not show any kind of correlation with the density recorded. However, the reason for the highest density, which was recorded in TvT9, might be due to the presence of it having the highest amount of bentonite clay (86.70-86.99%) in the formulation composition.

The biocontrol agent, *T. viride*, prefers to grow and function best at slightly acidic pH (Belal, 2008). Therefore, because the TvT1 composition has the lowest pH value of 7.16, it might be the best choice. This composition also exerted the lowest percentage of moisture content (7.55%, w w^-1^) and disintegration time (22.50 min). The composition of TvT1 was therefore selected for further experiments.

### Effect of tablet formulation (TvT) of *T. viride* against *F. oxysporum* on chickpea germination and wilting in pot experiment

**Figure 2:**
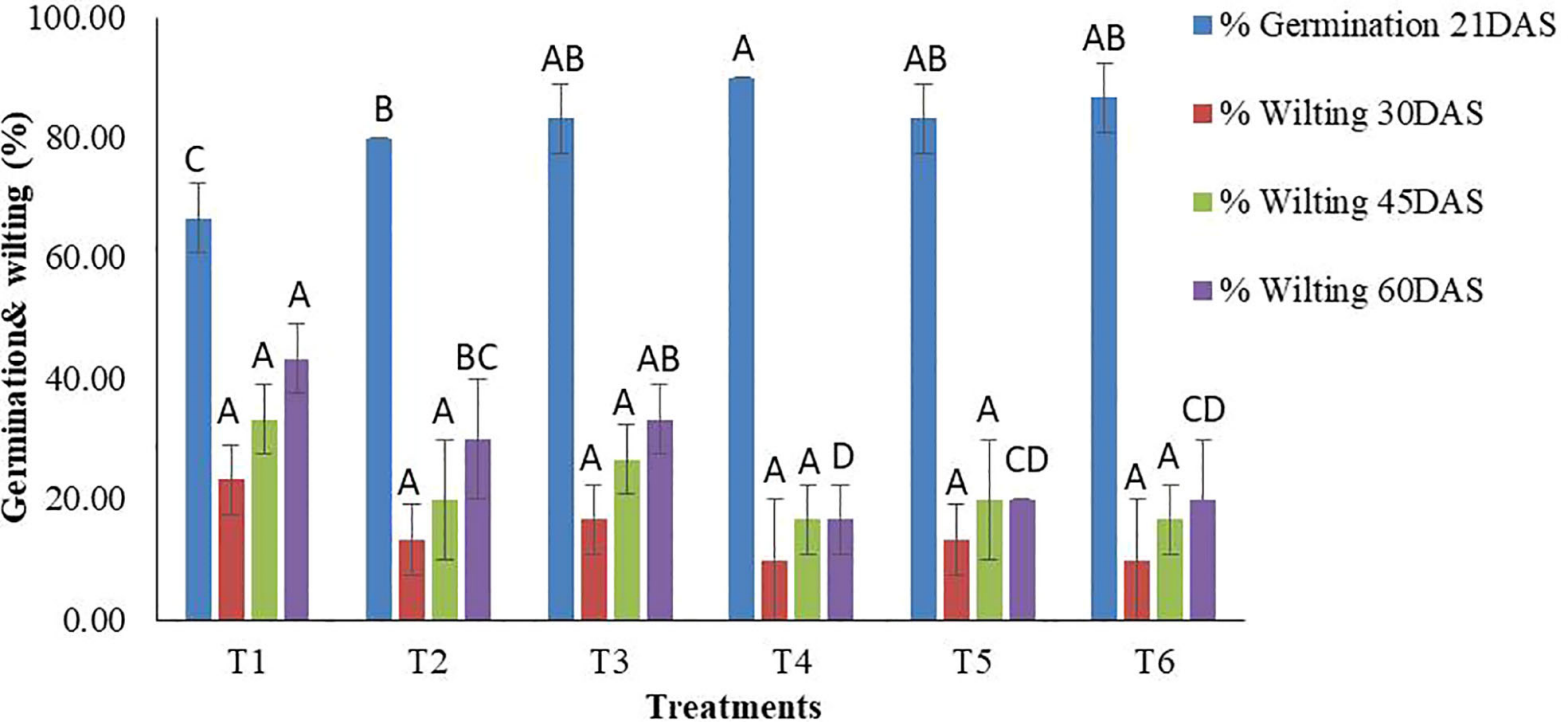
*In-vivo* assessment of percent seed germination and wilting of chickpea by TvT formulation of *T. viride* against pathogenic funus, *F. oxysporum* f. sp. *Ciceris.* (T1- Absolute control; T2- Carbendazim 50% WP; T3- Talc formulation; T4- TvT formulation at recommended dose; T5- TvT formulation at double dose; T6- TvT formulation at ½ of recommended dose). (For each parameter, treatment bars having at least one letter common are not statistically significant using Duncan’s Multiple Range Test at p<0.05, n=3, error bars represent standard deviations).

**Figure 3:**
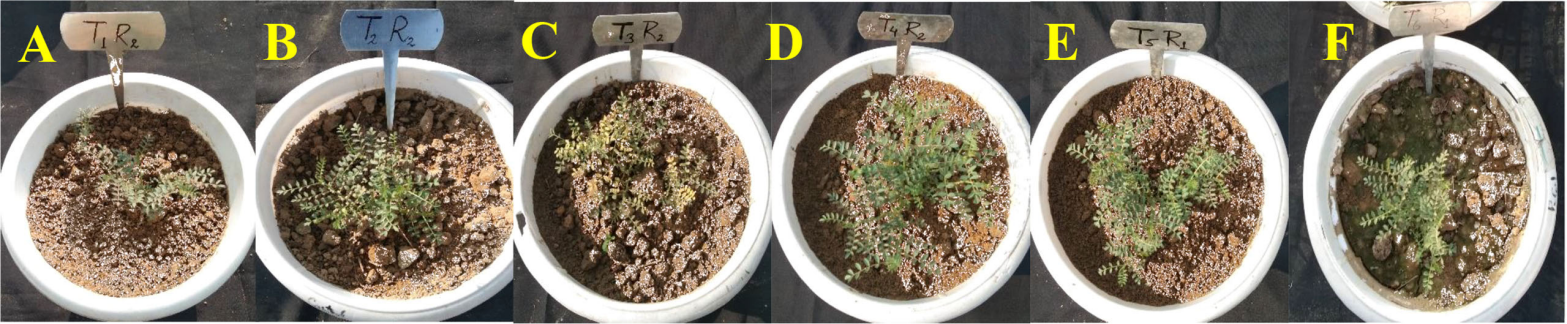
*In-vivo* assessment of wilt incidence in chickpea caused by wilt causing fungi, *F. oxysporum* f. sp. *ciceris* post application of TvT formulation of *T. viride*. (**A**- Absolute control; **B**- Carbendazim 50% WP; **C**- Talc formulation; **D**- TvT formulation at recommended dose; **E**- TvT formulation at double dose; **F**- TvT formulation at ½ of recommended dose).

Treatments were found to be significant (p<0.05) at all the replications under pot experiments for the germination percentage and wilting percentage at each time point (30, 45, and 60 DAS) (**Table S1**). As far as germination is concerned, a wide range of germination percentages were observed, ranging from 66.67-90.00%. The highest germination was recorded in T_4_ (TvT formulation at recommended dose level) while the lowest was in T_1_ (Absolute control). The TvT formulation at the three dose levels also performed significantly better than the commercial fungicide formulation treatment (T_2_) (**Figures 2**, **3**).

Wilting incidence was to be recorded lowest in the TvT formulation at the recommended dose level (T_4_), while it was at its highest in the absolute control (T_1_) for the three-time intervals (30, 45, 60 DAS). At 30 DAS, the highest wilting recorded was 23.33% (T_1_), while, 10.00% (T_4_ and T_6_) was the lowest. All the treatments were found to inhibit disease incidence more than that the commercial pesticide formulation (13.33%) as well as the talc-based formulation (16.67%) (**Figures 2**, **3**). At 45 DAS, the highest wilting recorded was 33.33% (T_1_) while 16.67% (T_4_) was the lowest. All the treatments were found to inhibit the disease incidence more than the commercial pesticide formulation (20.00%) as well as the talc formulation (26.67%). At 60 DAS, the highest wilting recorded was 43.33% (T_1_), while 16.67% (T_4_) was the lowest. All the treatments were found to inhibit the disease incidence higher than that of the commercial pesticide formulation (30.00%) as well as the talc-based formulation (33.33%).

### Bio-efficacy evaluation of TvP and TvT formulations on chickpea germination and wilting under field condition

**Figure 4:**
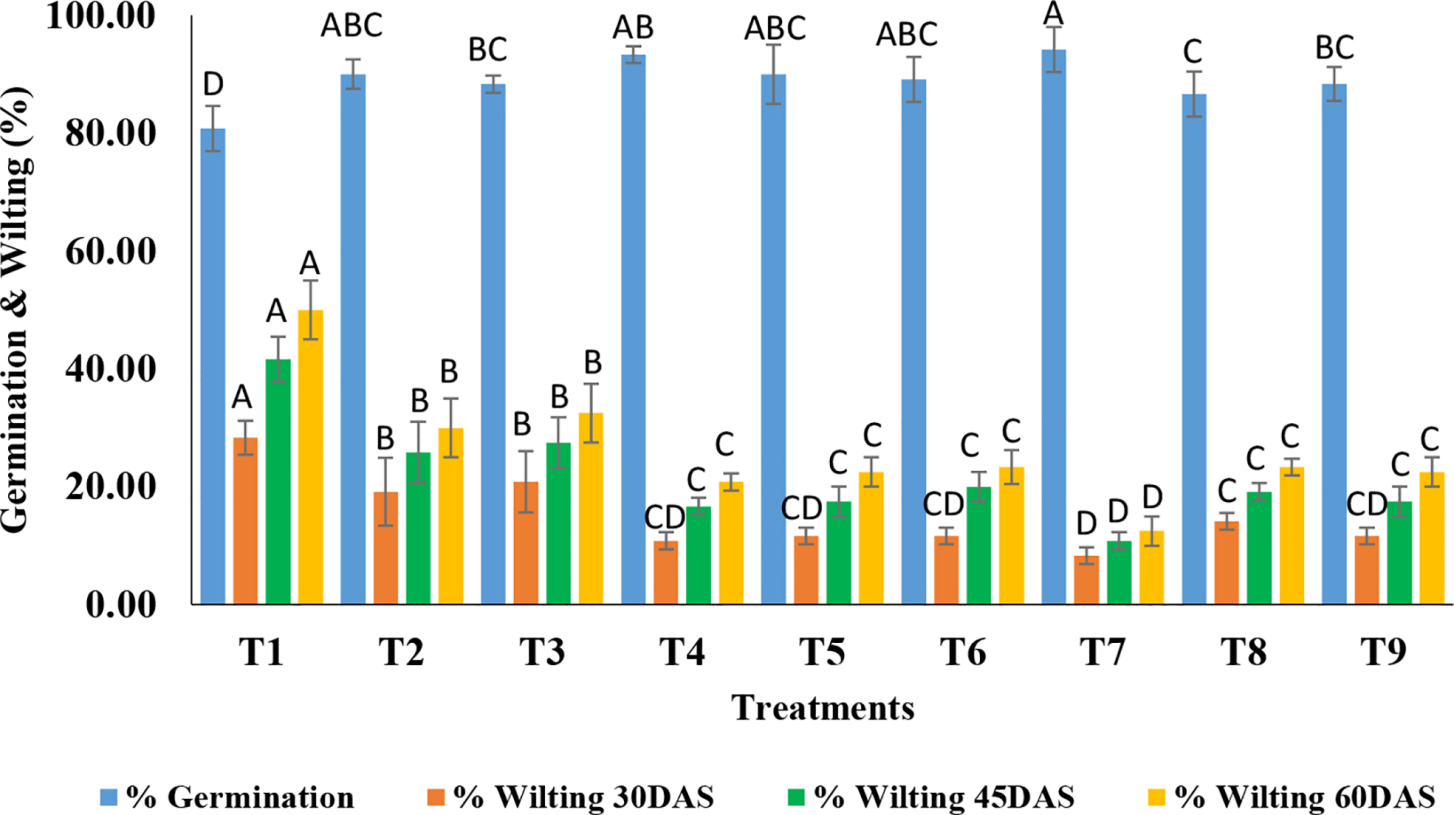
Effect of application of optimized TvT and TvP formulations against *F. oxysporum* f. sp. *ciceris* on percent seed germination and wilting of chickpea under field condition. (T1- Absolute control; T2- Carbendazim 50% WP; T3- Talc formulation; T4- TvT formulation at recommended dose; T5- TvT formulation at double dose; T6- TvT formulation at ½ of recommended dose; T7- TvP formulation at recommended dose; T8- TvP formulation at double dose; T9- TvP formulation at ½ of recommended dose.

**Figure 5:**
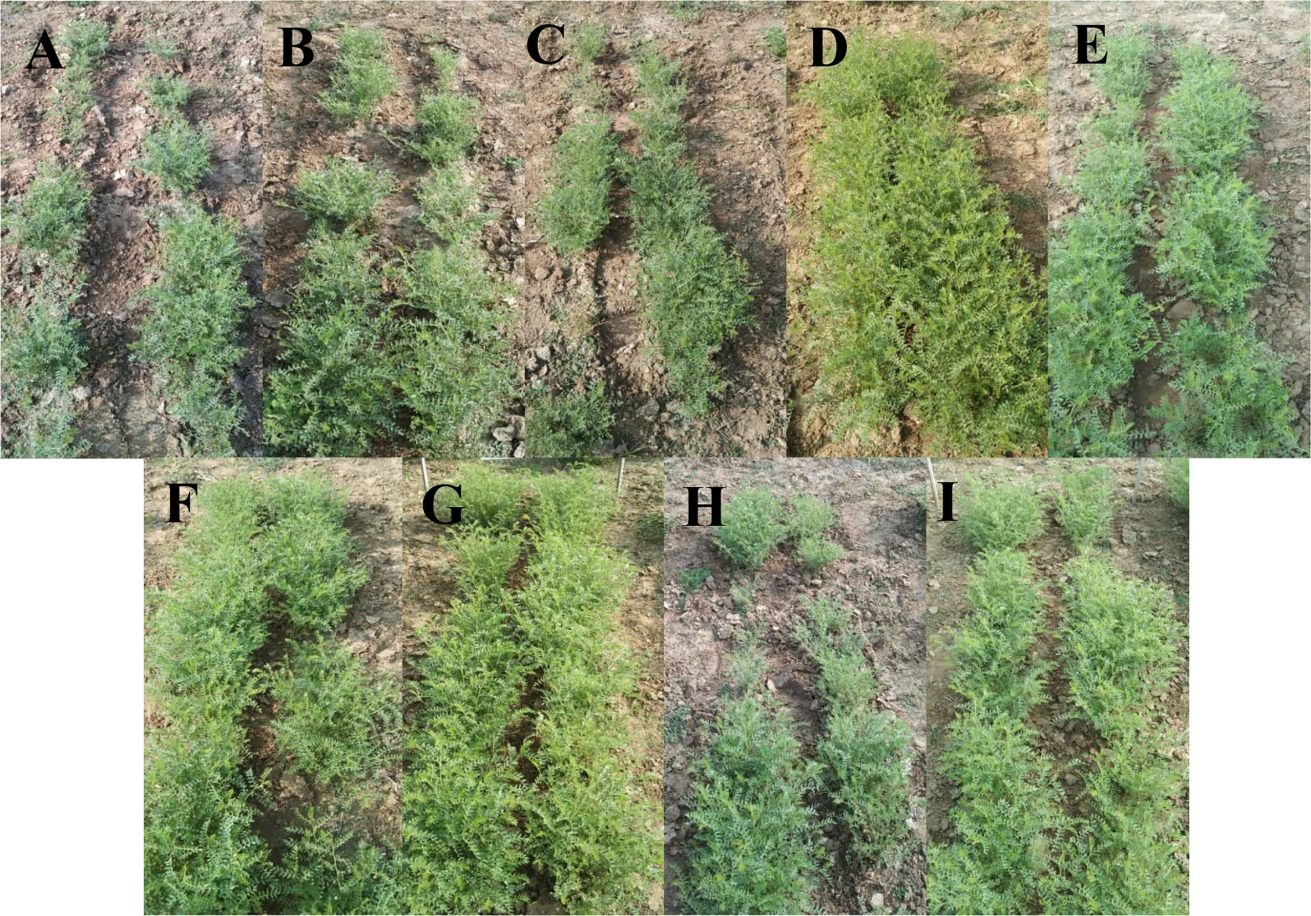
Field assessment of wilt incidence in chickpea caused by *F. oxysporum* f. sp. *ciceris* post application of TvT and TvP formulations of *T. viride.* (**A**- Absolute control; **B**- Carbendazim 50% WP; **C**- Talc formulation; **D**- TvT formulation at recommended dose; **E**- TvT formulation at double dose; **F**- TvT formulation at ½ of recommended dose; **G**- TvP formulation at recommended dose; **H**- TvP formulation at double dose; **I**- TvP formulation at ½ of recommended dose).

Treatments were found to be significant (p<0.05) under field conditions concerning the germination percentage and wilting percentage at the three-time intervals *i.e.* 30, 45, and 60 DAS (**Table S2**). As far as germination is concerned, a wide range of germination percentages were observed *i.e.* ranging from 80.83-94.17%. The highest germination was recorded in T_7_ (TvP formulation at the recommended dose level) while the lowest was in T_1_ (Absolute control) (**Figures 4**, **5**).

Wilting incidence was recorded as being lowest in powder formulation (TvP) at the recommended dose level (T_7_) while it was highest in the absolute control (T_1_) at the three time points time intervals (30, 45, 60 DAS). At 30 DAS, the highest wilting recorded was 28.33% (T_1_) while 8.33% (T_7_) was the lowest. All the treatments were found to inhibit the disease incidence significantly higher than that of the commercial pesticide formulation (19.17%) as well as the talc formulation (20.83%) (**Figure 4**). At 45 DAS, the highest wilting recorded was 41.67% (T_1_) while 10.83% (T_7_) was the lowest. All the treatments were found to inhibit the disease incidence significantly higher than the commercial fungicide, Carbendazim 50% WP formulation (25.83%) as well as the talc formulation (27.50%). At 60 DAS, the highest wilting recorded was 50.00% (T_1_) while 12.50% (T_7_) was the lowest. All the treatments were found to inhibit the disease incidence significantly more than Carbendazim (30.00%) as well as the talc formulation (32.50%).

### Effect of TvP and TvT formulations on chickpea yield parameters under field condition

Treatments were found to be significant (p<0.05) at all three replications under field conditions concerning the yield attributes *i.e.* root length, shoot length, shoot dry weight, and yield analysis. As shown in **Table 6**, the yield attributes follow the same trend as the germination and wilting studies. A wide range of differences in root length between the treatments were observed to be highest in T_7_ (24.9 cm) while the lowest was in T_1_ (10.03 cm). The biocontrol formulation-based treatment was the most effective compared to the commercial fungicide formulation (13.67 cm) and the talc-based formulation (16.00 cm). Similarly, shoot length ranged from T_1_ (32.43 cm) to T_7_ (44.40 cm) while no such effective difference was observed in the commercial pesticide formulation (38.53 cm) and the talc-based formulation (37.7 cm). Shoot dry weight was recorded in T_7_ (16.40 g) as being highest while T_1_ (8.23 g) was the lowest, meaning that all the treatments were superior over the talc-based formulation (11.37 g) and commercial pesticide formulation (11.73 g). Like other plant growth parameters, yield also followed the same trend, keeping the highest yield in T_7_ (21.29 q ha^-1^), whereas the lowest yield was obtained in T_1_ (15.16 q ha^-1^). The formulations developed in terms of yield were much more effective than the talc-based formulation (18.16 q ha^-1^) and commercial pesticide formulation (19.83 q ha^-1^).

**Table 6:** Effect of application of *T. viride*-based tablet (TvT) and powder (TvP) formulations against *F. oxysporum* f. sp. *ciceris* on plant growth and yield of chickpea in field conditions.

Treatment	Root length (cm)	Shoot length (cm)	Shoot dry weight (g)	Yield (q ha^-1^)
T1	10.03 ± 2.35^D^	32.43 ± 1.16^E^	8.23 ± 1.59^E^	15.16 ± 1.53^D^
T2	13.67 ± 2.22^C^	38.53 ± 2.00^D^	11.73 ± 1.37^CD^	19.83 ± 3.61^AB^
T3	16.00 ± 1.30^C^	37.70 ± 2.46^D^	11.37 ± 2.42^D^	18.16 ± 1.53^C^
T4	22.50 ± 0.65^B^	44.07 ± 0.25^AB^	15.30 ± 3.27^A^	20.46 ± 3.51^AB^
T5	21.57 ± 1.52^B^	42.47 ± 0.35^BC^	12.67 ± 2.58^BCD^	19.57 ± 2.00^BC^
T6	21.63 ± 1.16^B^	41.40 ± 0.36^C^	13.93 ± 2.60^ABC^	19.76 ± 2.08^AB^
T7	24.90 ± 0.80^A^	44.40 ± 0.46^A^	16.40 ± 1.42^A^	21.29 ± 2.52^A^
T8	21.50 ± 0.92^B^	42.80 ± 0.35^ABC^	14.30 ± 3.10^AB^	19.68 ± 3.06^BC^
T9	21.63 ± 0.21^B^	41.70 ± 0.53^C^	14.33 ± 2.95^AB^	19.93 ± 2.08^AB^
CV(%)	7.02	2.65	11.26	4.62

Means with at least one letter common are not statistically significant using DUNCAN’s Multiple Range Test at p<0.05, n=3.

(T1- Absolute control; T2- Carbendazim 50% WP; T3- Talc formulation; T4- TvT formulation at recommended dose; T5- TvT formulation at double dose; T6- TvT formulation at ½ of recommended dose; T7- TvP formulation at recommended dose; T8- TvP formulation at double dose; T9- TvP formulation at ½ of recommended dose).

## Discussion

*F. oxysporum* f. sp. *ciceris* is one the most potent yield-limiting factors of chickpea (*Cicer arietinum* L.) worldwide. It enters through the roots and the germ tube enters plant epidermal cells. Eventually, the hyphae expand to the root cortical region and invade the xylem vessels, preventing water and other critical solutes from being transported upward and causing wilt. Additionally, the saprophytic fungus can live in soil or debris for up to 6 years and cause a reduction in yield (Caballo et al., 2019). Therefore, extensive research has been undertaken with an economical and feasible approach to manage the disease and a biological control-based approach has been found suitable (Dubey et al., 2007). Several efforts toward the management of soil or seed-borne diseases support the *Trichoderma* spp. as the best antagonist of pathogens (Dubey, 2002; Poddar et al., 2004). The antagonism caused by *Trichoderma* against *Fusarium* spp. is due to the coiling of antagonistic fungal hyphae, thereby causing lysis (Kumar and Dubey, 2001; Naglot et al., 2015; El-Debaiky, 2017; Chen et al., 2021). In the present work, the selection study of the best performing strain of *T. viride* revealed the efficacy of the strain ITCC 7764 as the most efficient. GC-MS analysis of the VOCs released by the fungal strain showed the highest content of octan-3-one (13.92%), 3-octanol (10.57%), and 1-octen-3-ol (9.40%). These compounds did not appear in the less potential strains, *T. viride* 8276 and *T. viride* 7847, where long-chain hydrocarbon contributed maximum in their respective composition. According to some of the studies, several species of *Trichoderma* have been found to generate different chemicals that prevent the growth and development of pathogenic fungi and also mycoparasitize them in crops, such as *T. harzianum* on *R. solani* (Poveda, 2021). In another investigation by Kumar et al. (2019), *T. viride* and *T. harzianum* exhibited inhibition zones of 70.10% and 76.90% against the FOC strain of *F. oxysporum*, respectively.

Based on the screening study, the strain ITCC 7764 was selected as the bioagent to develop *T. viride-*based biocontrol formulations. Two types of biocontrol formulations of *T. viride* strain ITCC 7764, TvP for seed treatment and TvT for direct application, have been developed in the present study with different compositions of biopolymer, optimized xerogel, neem leaf powder, and clay. The present study undertook the selection of formulation auxiliaries critically to prepare the powder and tablet matrix suitable for bioagent survival and bioefficacy. Clay is generally used in formulations as a filler material (Le Bras and Bourbigot, 1996). Biopolymer has been extensively used in the development of biocontrol formulations due to their nontoxicity, wider availability, and cost-effectiveness (Mukhopadhyay et al., 2019; Meena et al., 2020; Oszust et al., 2021). The presence of xerogel in the formulation matrix may provide a sustained moisture-enriched environment during storage (Mukhopadhyay et al., 2020) and supply moisture to the crop after getting released to the field through formulation application (Akhter et al., 2004; Prakash et al., 2021). Moreover, the neem leaf powder, which was taken as an excipient of the prepared formulations expressed, no negative impact on test antagonistic fungi, *T. viride*, expressing its compatibility and suitability as an adjuvant of the formulations. The neem leaf powder expressed 12.80% and 34.90% inhibition of *F. oxysporum* f. sp. *ciceris* singly and in combination with *T. viride* at the concentration present in the recommended dose of the formulation. A similar finding was observed by Patil et al. (2018), where the application of neem leaves at the rate of 30 g per pot revealed a lower percentage of damping-off caused by *F. oxysporum* f. sp. *cucumerinum* as compared to bavistin and untreated inoculated check of the fungus alone in cucumber at 30 DAS. The azadirachtin content has been reported to be 0.0244 percent (w w^-1^) in neem leaves (Radhakrishnan et al., 2010). In another study by Prashanth and Krishnaiah (2014), the alkaloids, reducing sugars, saponins, and other phytochemicals were detected in the ethanolic and aqueous extracts of neem leaves. Numerous other substances, including acetyloxy acetic acid, germanicol, 1,3-diphenyl-2-azafluorene, phytol, and hydroxy pivalic acid, were also detected in the ethanolic extract of the neem leaves by GC-MS. Singh et al. (2010) prepared a controlled release fungicidal formulation of thiram by employing alginate beads and neem leaf powder.

The various physicochemical parameters of the developed formulations were also analyzed using standard CIPAC guidelines. For the selection of the optimized formulations, the pH of the formulations was selected as the most important criterion. The biocontrol agent, *T. viride*, has been previously reported to grow best and function at pH 6.50 (Belal, 2008). Therefore, the formulations TvP2 (pH: 6.50) and TvT1 (pH 7.16) were selected for further experimentation. Immobilizing wet or dry biomass inside cross-linked polymers like alginate and carrageenan is one of the most current techniques for generating biocontrol formulations (Saberi-Riseh et al., 2021). Bejarano and Puopolo (2020) reported the formulation of pellets by dry and wet immobilization of the biocontrol agents. For the entrapment of bio-agent conidia, sodium alginate, aluminum silicate, and sabudana powder were utilized by Dubey et al. (2009) to develop *Trichoderma*-based formulations for soil application. Furthermore, granular and liquid formulations of *Trichoderma* have been developed for the control of *R. solani* (Herrera et al., 2020).

The wilt disease caused by *F. oxysporum* is one the most prominent yield-limiting diseases affecting chickpea worldwide. Yield losses have been recorded from 10 to 100%, varying with the differences in the prevailing climatic conditions (Jiménez-Díaz et al., 2015; Sharma et al., 2019). Fungal soil-borne diseases have been hard to manage, especially when the pathogen adopts some resistant means of survival such as chlamydospores or sclerotia, enabling the pathogen to infect the crop and soil for up to several years (Maitlo et al., 2019). Being soil-borne, chemical control measures are applied several times for controlling the wilt in chickpea crops. However, the biological control based approach is more suitable compared to chemical methods (Dubey et al., 2007). In the present findings, the observed wilting percentage was only 16.67% after 60 DAS at the recommended dose of the formulation TvT1 under *in vivo* conditions. Moreover, the bioefficacy of the TvT1 formulation was significantly higher as compared to the carbendazim and *Trichoderma* talc-based formulation.

Several efforts toward the management of soil or seed-borne diseases support the *T. viride* and *T. harzianum* to be the best antagonists of pathogens (Dubey, 2002; Poddar et al., 2004). The same biocontrol agent has been assessed against *Fusarium* species, resulting in the production of various volatiles and non-volatiles having antagonistic potential towards the pathogen and resulting in its suppression (Kumar and Dubey, 2001; Bubici et al., 2019). A bioefficacy study under field conditions expressed the superior performance of TvP formulation as compared to the Carbendazim and talc-based biocontrol formulation. Moreover, at 60 DAS, the highest wilting recorded was 50.00% in the negative control while 12.50% was the lowest in the TvP formulation at the recommended dose. In another *in vitro* study, the biopolymeric hydrogel-based formulation composition containing *Trichoderma* sp. spores and/or mycelia was found to be more effective at combating *R. solani* compared to *Trichoderma* sp. alone (Mondal et al., 2020). A similar observation was also reported by Animisha et al. (2012), suggesting only 19% occurrence of wilt disease after application of *T. viride* compared to the 21% incidence observed on treatment with Carbendazim.

Different herbicide tablets have already been developed for sustained release applications (Shahena et al., 2020). However, the preparation of antagonistic fungi-loaded tablet formulations for direct applications to the field employed in this study is novel. The performance of a tablet formulation depends on several factors such as dissolution, tablet breaking force, disintegration, the homogeneity of composition, etc. (Akseli et al., 2017) Similarly, the effect of *Trichoderma* based formulation depends on different factors namely p^H^, moisture content, inoculum concentration, storage temperature, inoculum age, and the incubation period (Mulatu et al., 2021). Therefore, integrating knowledge of material science and fungal physiological behavior is paramount for the development of antagonistic fungi-based tablet bioformulations. In the present investigation, the TvT formulation showed improved bioefficacy compared to the positive controls (Carbendazim 50% WP and talc formulation), but it expressed inferior performance compared to TvP under field conditions. This might be due to the presence of alkaline formulation components resulting in the p^H^ of the matrix being higher than 6.50, which was not conducive to the growth and functioning of the biocontrol agent. The high disintegration time (22.5 min) might also be responsible for the slower release and buildup of antagonistic fungal growth in the soil. Moreover, the proximity to the seeds being further away in the case of the TvT formulation might account for its worse performance in terms of wilting incidence compared to the TvP formulation.

## Conclusion

In total, five different strains of *T. viride* (ITCC 6889, ITCC 7204, ITCC 7764, ITCC 7847, and ITCC 8276) were screened against *F. oxysporum* f. sp. *ciceris*, causing devastating wilt disease in chickpea. The selection of the most effective *Trichoderma* strain for the development of biocontrol formulations was based on *in-vitro* inhibition tests using volatile and non-volatile assays. The best strain ITCC-7764 recorded an inhibition of 26.45% and 74.45%, respectively. Furthermore, strain TV-3 (ITCC 7764) was used to formulate powder for seed treatment (TvP) and tablet (TvT) formulations applied at the three dose levels (recommended dose, double of the recommended dose, and half of the recommended dose), the effectivity of which were assessed in terms of germination and wilting percentage for Carbendazim as commercial formulation and talc-based formulations under field conditions. Under field conditions, the powder formulation TvP for seed treatment at the recommended dose was recorded to be more effective against the pathogen than a tablet (TvT) applied treatment, though both recorded superior performance to Carbendazim 50% WP and talc based formulation. Overall, the research undertaken reports two different types of bio-control formulations (TvP and TvT) of *T. viride* as effective environmentally sustainable options for managing wilting disease in chickpeas. These findings on the novel powder and tablet formulations of *T. viride* will be taken forward to validate their product performance under shelf-life assessment and integrated disease management programs.

## Supplementary information

### Xerogel preparation method

An *in-situ* solution polymerization was used to synthesize the biopolymeric agri-waste reinforced superabsorbent composites taking various ratios of backbone, agriwaste, monomer, cross-linker and initiator in a definite volume of water at a particular temperature. The alkali was used after gel formation. Gel was suitably treated to attain pH of 7.0 and dried to get xerogel.

## Data availability statement

The original contributions presented in the study are included in the article/**Supplementary Material**. Further inquiries can be directed to the corresponding author.

## Author contributions

PP, AM, RK, AK and PS conducted the investigation. NP, AD and DK validated the study. AS and RA provided resources. AS conceptualized the study. PP, AM, RK and AK prepared the original draft. AS, CB and TB supervised the experiment. All authors contributed to the article and approved the submitted version.

## Acknowledgments

The authors thank the Director of the Indian Council of Agricultural Research-Indian Agricultural Research Institute (ICAR-IARI), New Delhi, India.

## Conflict of interest

The authors declare that the research was conducted in the absence of any commercial or financial relationships that could be construed as a potential conflict of interest.

## Publisher’s note

All claims expressed in this article are solely those of the authors and do not necessarily represent those of their affiliated organizations, or those of the publisher, the editors and the reviewers. Any product that may be evaluated in this article, or claim that may be made by its manufacturer, is not guaranteed or endorsed by the publisher.

## Supplementary material

Supplementary Figure 1 Bioefficacy assessment of different strains of *T. viride* against *F. oxysporum* f. sp. *ciceris* using volatile method

Supplementary Figure 2 Bioefficacy assessment of different strains of *T. viride* against *F. oxysporum* f. sp. *ciceris* using non-volatile method

Supplementary Table 1 Effect of Application of *T. viride*-based Tablet (TvT) Formulation against *F. oxysporum* f. sp. *ciceris* on Seed Germination and Wilting Incidence on Chickpea *in-vivo*

Supplementary Table 2 Effect of Application of *T. viride*-based tablet (TvT) and Powder (TvP) Formulations against *F. oxysporum* f. sp. *ciceris* on Seed Germination and Wilting Incidence on Chickpea in Field Conditions
