# Supplementary material for: Performance appraisal of Trichoderma viride based novel tablet and powder formulations for management of Fusarium wilt disease in chickpea
Source: Front Plant Sci. 2022 Oct 7;13:990392. doi: 10.3389/fpls.2022.990392 (PMC9585344; doi:10.3389/fpls.2022.990392)
Supplement: Supplementary Figure 1 — Bioefficacy assessment of different strains of T. viride against F. oxysporum f. sp. ciceris using volatile method [file DataSheet_1.docx]

**SUPPLEMENTARY INFORMATION**

**Supplementary Figure Legends:**

**Fig. S1.** Bioefficacy assessment of different strains of *T. viride* against *F. oxysporum* f. sp. *ciceris* using volatile method

**Fig. S2.** Bioefficacy assessment of different strains of *T. viride* against *F. oxysporum* f. sp. *ciceris* using non-volatile method

**
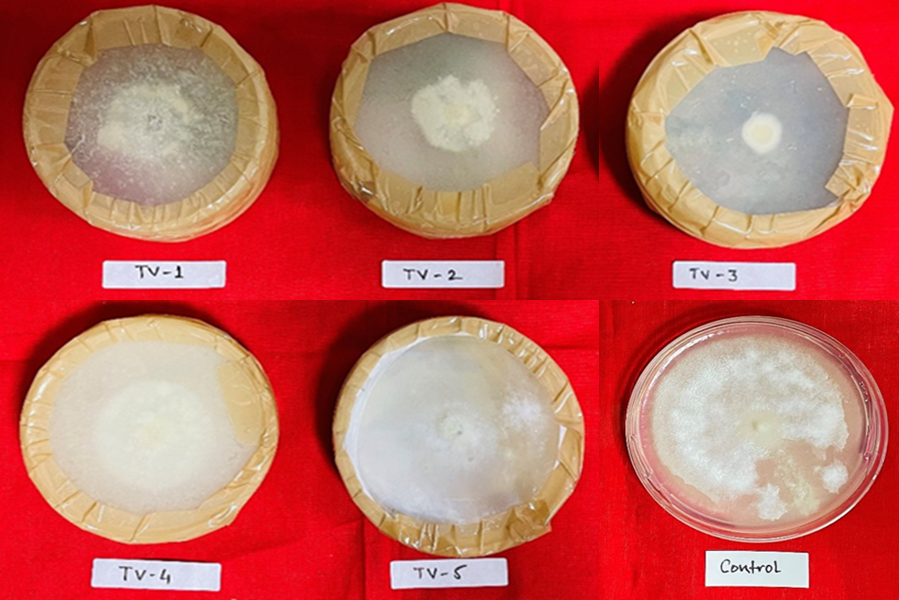
**

**Fig. S1.**

**
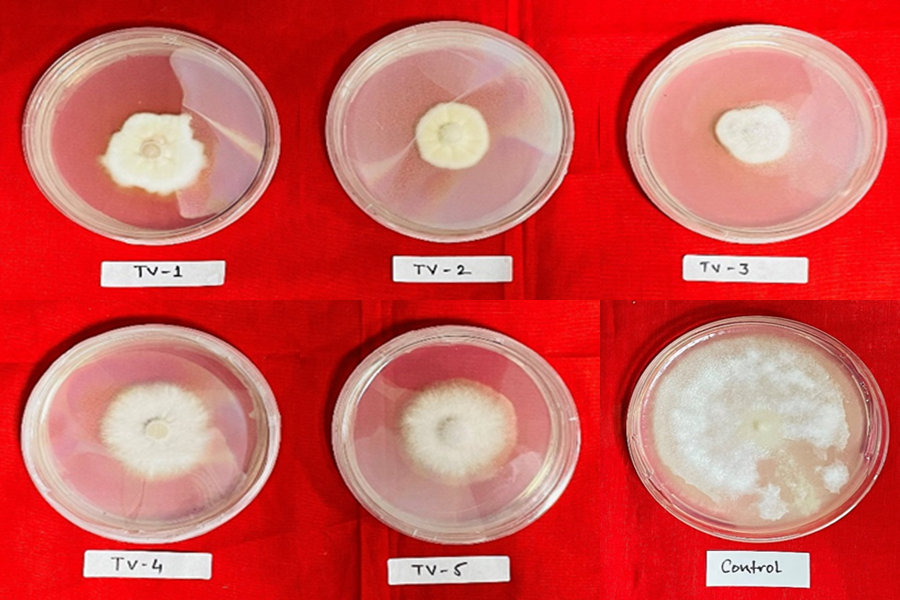
**

**Fig. S2.**

**Table S1.** Effect of Application of *T. viride*-based Tablet (TvT) Formulation against *F. oxysporum* f. sp. *ciceris* on Seed Germination and Wilting Incidence on Chickpea *in-vivo*

| **Treatment** | **Germinated**  **plants 21 DAS** | **Wilted plants 30 DAS** | **Wilted plants 45 DAS** | **Wilted plants 60 DAS** |
| --- | --- | --- | --- | --- |
| T1 | 6.67$\pm$0.58^D^ | 2.33$\pm$0.58^A^ | 3.33$\pm$0.58^A^ | 4.33$\pm$0.58^A^ |
| T2 | 8.00$\pm$0.00^C^ | 1.33$\pm$0.58^ABC^ | 2.00$\pm$1.00^BC^ | 3.00$\pm$1.00^BC^ |
| T3 | 8.33$\pm$0.58^BC^ | 1.67$\pm$0.58^AB^ | 2.67$\pm$0.58^AB^ | 3.33$\pm$0.58^AB^ |
| T4 | 9.00$\pm$0.00^A^ | 1.00$\pm$1.00^BC^ | 1.67$\pm$0.58^BCD^ | 1.67$\pm$0.58^D^ |
| T5 | 8.33$\pm$0.58^BC^ | 1.33$\pm$0.58^ABC^ | 2.00$\pm$1.00^BC^ | 2.00$\pm$0.00^CD^ |
| T6 | 8.67$\pm$0.58^ABC^ | 1.00$\pm$1.00^BC^ | 1.67$\pm$0.58^BCD^ | 2.00$\pm$1.00^CD^ |
| CV(%) | 4.63 | 49.17 | 36.37 | 27.98 |

*Means with at least one letter common are not statistically significant using DUNCAN's Multiple Range Test at p<0.05, n=3

(T1- Absolute control; T2- Carbendazim 50% WP; T3- Talc formulation; T4- TvT formulation at recommended dose; T5- TvT formulation at double dose; T6- TvT formulation at ½ of recommended dose)

**Table S2.** Effect of Application of *T. viride*-based tablet (TvT) and Powder (TvP) Formulations against *F. oxysporum* f. sp. *ciceris* on Seed Germination and Wilting Incidence on Chickpea in Field Conditions

| **Treatment** | **Germinated**  **plants**  **21 DAS** | **Wilted plants**  **30 DAS** | **Wilted plants**  **45 DAS** | | **Wilted plants**  **60 DAS** |
| --- | --- | --- | --- | --- | --- |
| T1 | 31.00$\pm$1.53^E^ | 11.33$\pm$1.15^A^ | 16.67$\pm$1.53^A^ | 20.00$\pm$2.00^A^ | |
| T2 | 35.00$\pm$1.00^CD^ | 7.67$\pm$2.31^B^ | 10.33$\pm$2.08^B^ | 12.00$\pm$2.00^B^ | |
| T3 | 35.00$\pm$0.58^CD^ | 8.33$\pm$2.08^B^ | 11.00$\pm$1.73^B^ | 13.00$\pm$2.00^B^ | |
| T4 | 36.67$\pm$0.58^AB^ | 4.33$\pm$0.58^CD^ | 6.67$\pm$0.58^C^ | 8.33$\pm$0.58^C^ | |
| T5 | 35.00$\pm$2.00^CD^ | 4.67$\pm$0.58^CD^ | 7.00$\pm$1.00^C^ | 9.00$\pm$1.00^C^ | |
| T6 | 34.33$\pm$1.53^D^ | 4.67$\pm$0.58^CD^ | 8.00$\pm$1.00^C^ | 9.33$\pm$1.15^C^ | |
| T7 | 37.00$\pm$1.53^A^ | 3.33$\pm$0.58^D^ | 4.33$\pm$0.58^D^ | 5.00$\pm$1.00^D^ | |
| T8 | 35.67$\pm$1.15^BC^ | 5.67$\pm$0.58^C^ | 7.67$\pm$0.58^C^ | 9.33$\pm$0.58^C^ | |
| T9 | 35.67$\pm$^BC^ | 4.67$\pm$0.58^CD^ | 7.00$\pm$1.00^C^ | 9.00$\pm$1.00^C^ | |
| CV(%) | 1.67 | 18.58 | 14.37 | 12.83 | |

*Means with at least one letter common are not statistically significant using DUNCAN's Multiple Range Test at p<0.05, n=3

(T1- Absolute control; T2- Carbendazim 50% WP; T3- Talc formulation; T4- TvT formulation at recommended dose; T5- TvT formulation at double dose; T6- TvT formulation at ½ of recommended dose; T7- TvP formulation at recommended dose; T8- TvP formulation at double dose; T9- TvP formulation at ½ of recommended dose)
